# Supplementary material for: A user-friendly herbicide derived from photo-responsive supramolecular vesicles
Source: Nat Commun. 2018 Jul 27;9:2967. doi: 10.1038/s41467-018-05437-5 (PMC6063903; doi:10.1038/s41467-018-05437-5)
Supplement: Supplementary file 1 — Supplementary Information [file 41467_2018_5437_MOESM1_ESM.pdf]

Supplementary Information

A user-friendly herbicide derived from photo-responsive supramolecular vesicles

Gao et al.

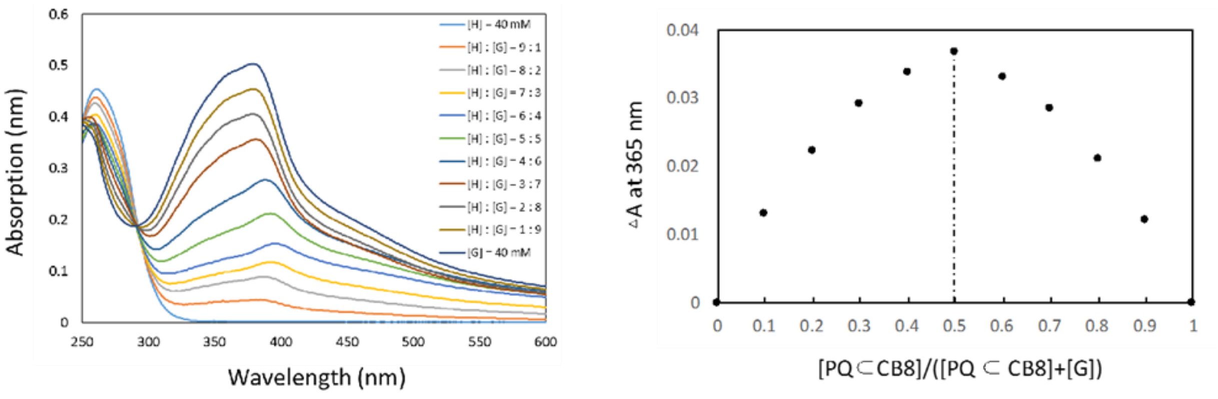

Supplementary Figure 1. **Complexation of CB[8], PQ and *trans*-G.** Job plot of (trans-G)-PQ⊂CB[8] monitored by UV-visible spectroscopy.

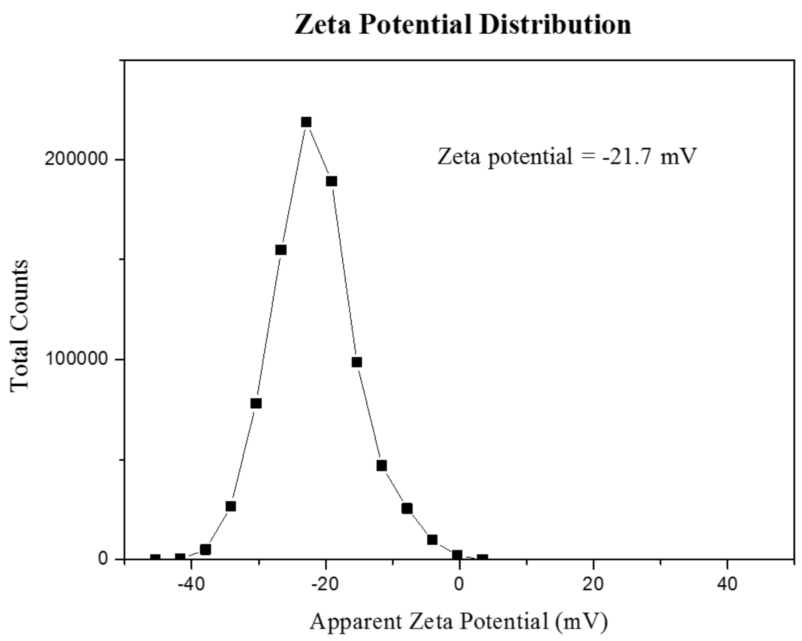

Supplementary Figure 2. Zeta potential of the vesicles. DLS showed that the zeta potential of the vesicles was -21.7 mV, indicating that the vesicles had a good colloidal stability because of the repulsive effect.

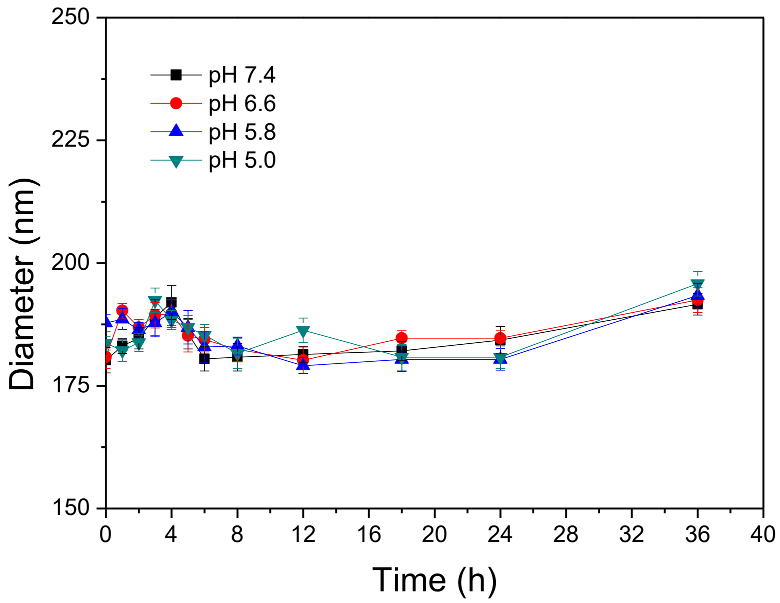

Supplementary Figure 3. Stability of the vesicles under different pH conditions. The vesicles were placed in PBS with different pH values, and their sizes were determined by DLS over 36-h follow-up. The experiments were replicated for 3 times independently.

(a)

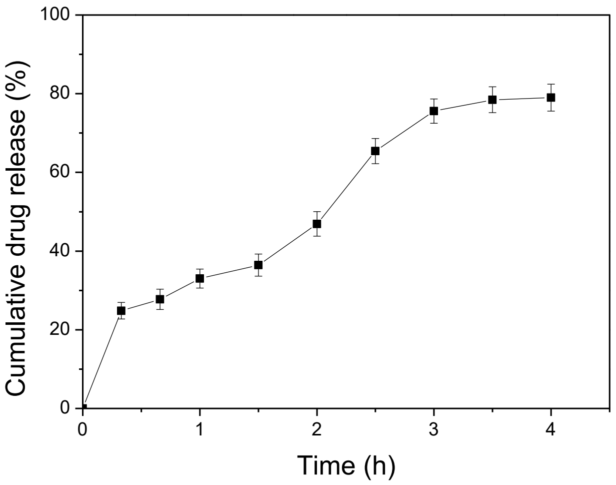

(b)

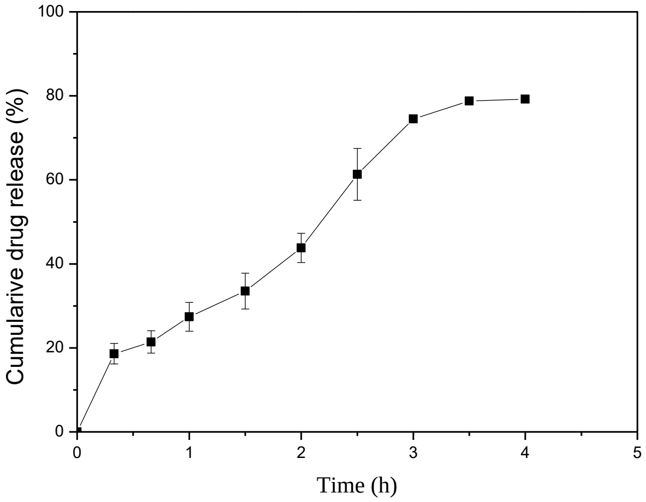

Supplementary Figure 4. Drug release profile. **(a)** Drug release profile under irradiation of natural sunlight (Macau, March 3, 2018, Sunny) of PQ-loaded vesicles after storage in the dark for 210 days. **(b)** Drug release profile from diquat-loaded vesicles under natural sunlight (Macau, March 22, 2018, Sunny). The experiments were replicated for 3 times independently.

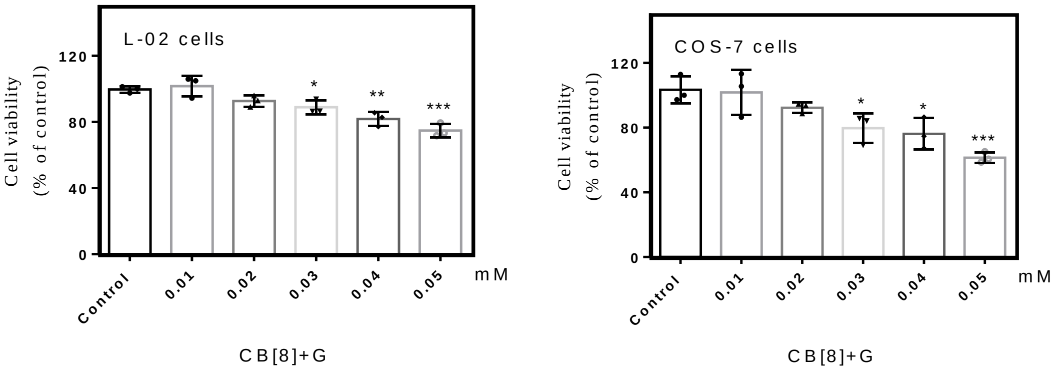

Supplementary Figure 5. Cytotoxicity of CB[8]+G. L-02 and COS-7 cells were incubated with various concentrations of raw materials (CB[8]+G) for 3 days. The error bars correspond to the S.D. (n = 3). *$P \leq 0.05$, **$P \leq 0.01$ and ***$P \leq 0.001$ determined by one-way ANOVA.

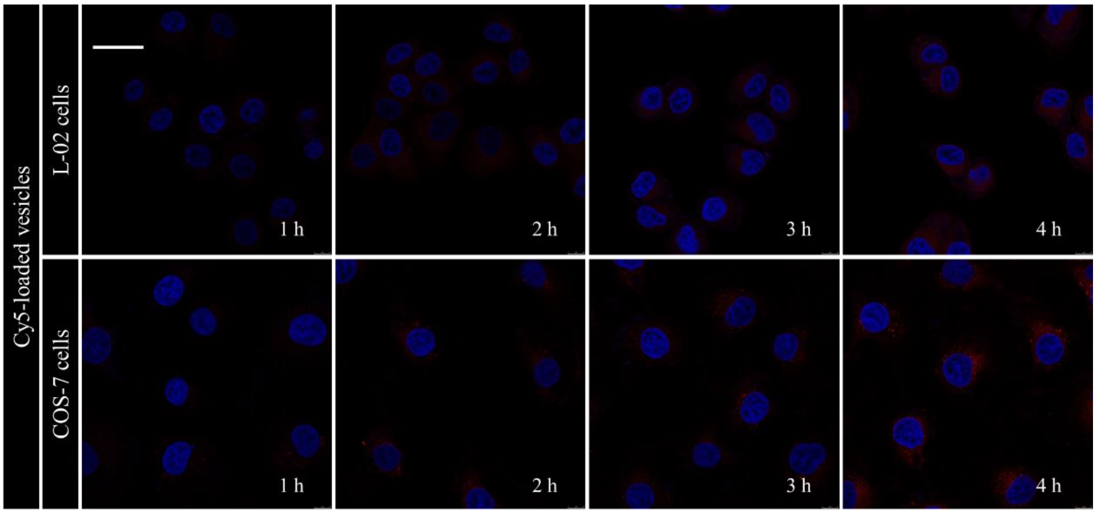

Supplementary Figure 6. Intracellular uptake of Cy5-loaded vesicles. Fluorescent microscopic images of L-02 cells and COS-7 cells incubated with media containing Cy5-loaded vesicles for 1, 2, 3 and 4 h. Scale bar: 100 μm.

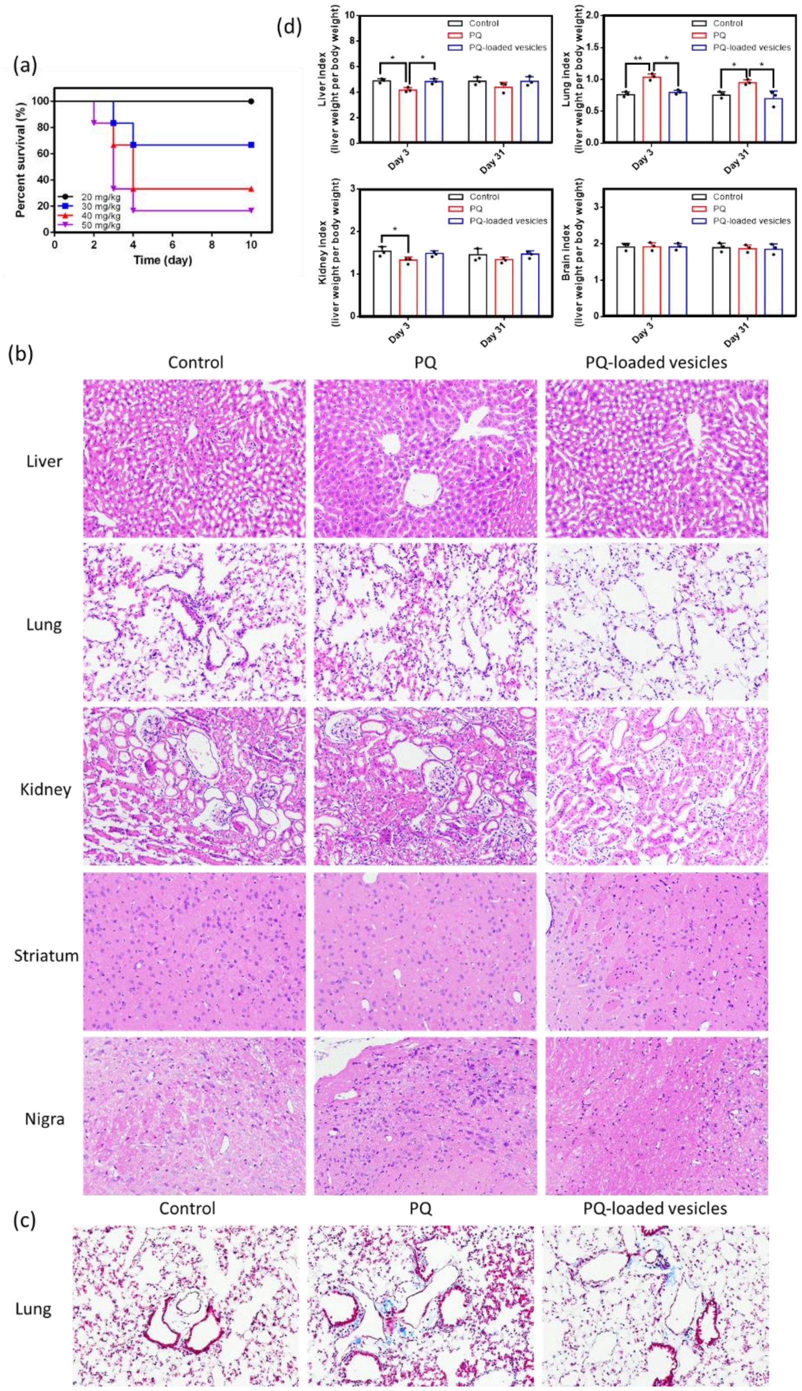

Supplementary Figure 7. Safety evaluation on mouse model. (**a**) Mice were intraperitoneally administrated with PQ at different dose (20mg kg^{-1}, 30 mg kg^{-1}, 40 mg kg^{-1} and 50 mg kg^{-1}, n = 6 in each group). (**b**) HE staining on the liver, lung, kidney, striatum and nigra in the mice with a single-dose administration with different formulations (PQ dose of 30 mg kg^{-1}). The organs were harvested on Day 31 post dose administration. (**c**) Masson's trichrome staining on the lung in mice Day 31 post dose administration (PQ dose of 30 mg kg^{-1}). (**d**) The organ index of the liver, lung, kidney and brain, on Day 3 and Day 31, respectively, in the mice administered with 30 mg kg^{-1} PQ on Day 1. The error bars correspond to the S.D. (n = 3). *$P \leq 0.05$, **$P \leq 0.01$ and ***$P \leq 0.001$ determined by one-way ANOVA.

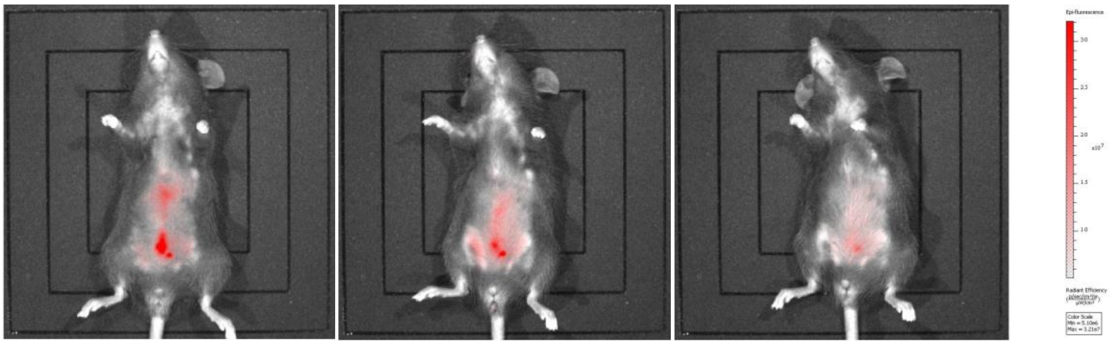

1st day 3rd day 6th day

Supplementary Figure 8. Distribution of Cy7.5 labeled nanoparticles in mice. The mice were intraperitoneally administrated with Cy7.5-loaded vesicles and subsequently imaged by a living imaging system (IVIS Lumina III In Vivo Imaging System) at predesignated time points.

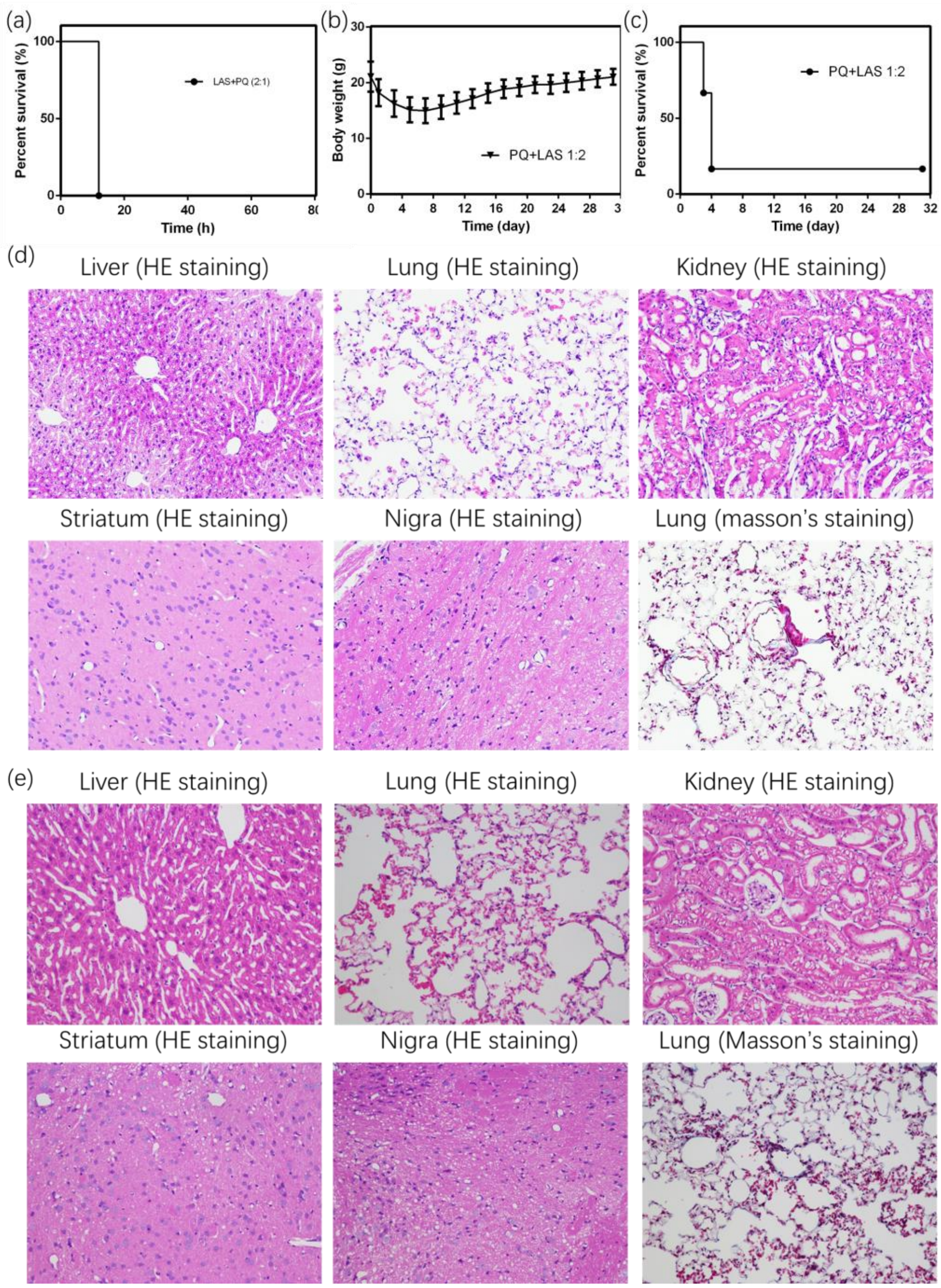

Supplementary Figure 9. Systemic safety evaluation of a previously reported PQ formulation (PQ:LAS, 1: 2) (Pest Manag. Sci., 2013, 69, 553-558) on mouse model. (**a**) The survival rate of 30-dpf zebrafish treated with PQ:LAS (1:2) at PQ dose of 2 mM and LAS dose of 4 mM (n = 10 in each group). (**b**) The changes of body weight of the mice intraperitoneally administered with PQ+LAS (1:2) at PQ dose of 20 mg kg^{-1}. The error bars correspond to the S.D. (n = 6). (**c**) The mortality rate of the mice intraperitoneally administered with PQ+LAS (at PQ dose of 40 mg kg^{-1}) (n = 6 in each group). (**d**) Histological study on the liver, lung, kidney, striatum and nigra in these mice on Day 3, after administration with PQ+LAS at 30 mg kg^{-1}. (**e**) Histological study on the liver, lung, kidney, striatum and nigra in the mice on Day 31, after administration with PQ+LAS at 30 mg kg^{-1}.

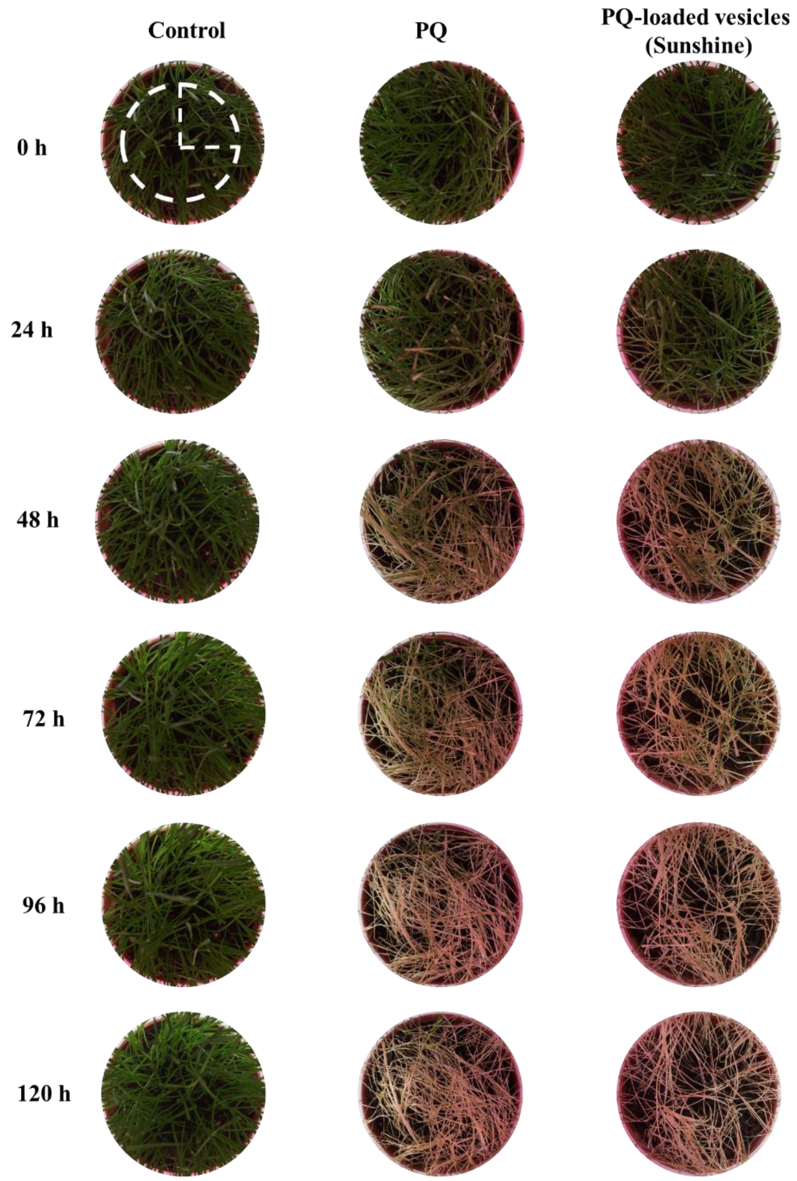

Supplementary Figure 10. Weed control efficacy of free PQ and PQ loaded photo-responsive vesicles under natural sunlight. Foliar application of control (water), PQ and PQ-loaded vesicles and monitored under natural sunlight in a natural setting, at a single dose of 2 mg mL^{-1}. Diameter of the white-dashed circle: 6 cm.

Supplementary Note.

The herbicidal experiment was conducted in Macau from March 22, 2018 to March 27, 2018, and the weather condition was listed in the following chart.

**Date**	March 22, 2018	March 23, 2018	March 24, 2018
**Weather condition**	Sunny	Sunny	Passing clouds
**Date**	March 25, 2018	March 26, 2018	March 27, 2018
**Weather condition**	Scattered clouds	Passing clouds	Partly cloudy

a

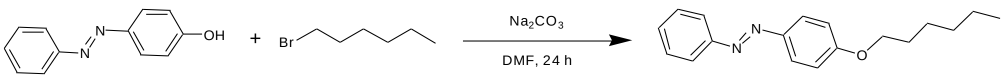

b

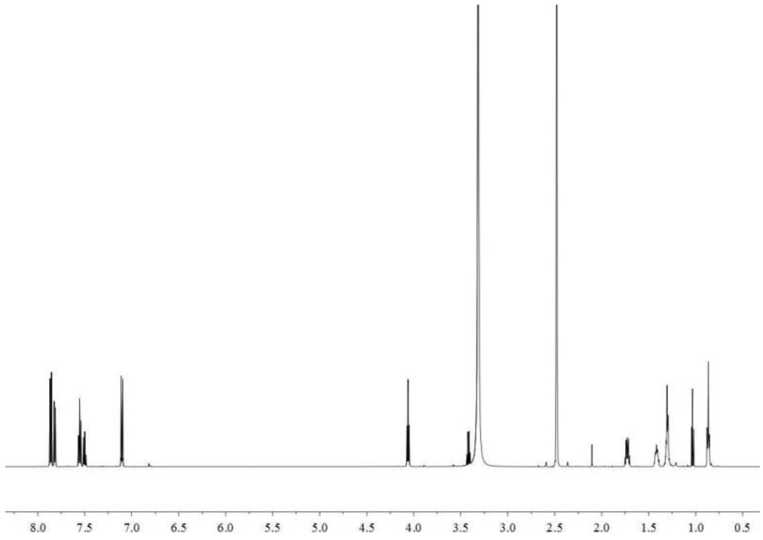

c

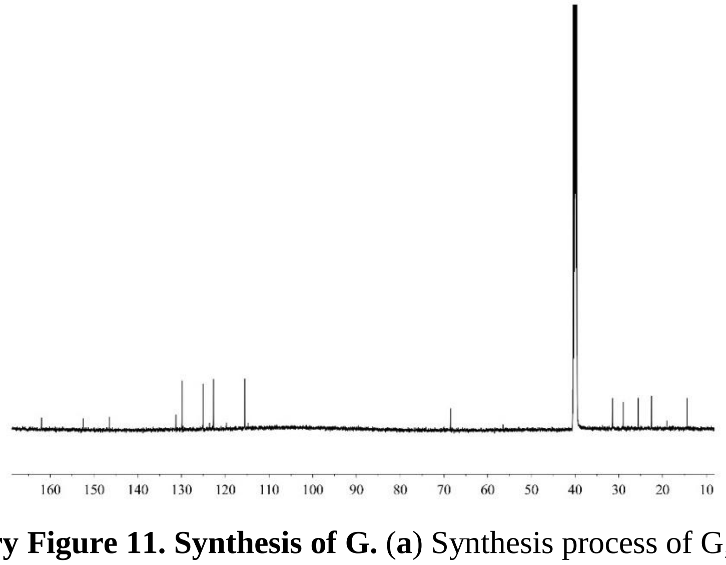

Supplementary Figure 11. Synthesis of G. (**a**) Synthesis process of G; (**b**) ^{1}H NMR (600 MHz) spectrum of G in D_2O; (**c**) ^{13}C NMR spectrum of G in DMSO-d_6.

Supplementary Table 1. Stability of the vesicles.

(a) The MD (Mean Diameter) and PDI (polydispersity index) of vesicles incubated in PBS under dark conditions were determined by DLS before and after storage for 3 days and 210 days.

Time (day)	Vesicles	
	MD (nm)	PDI
0	187.8	0.158
3	208.1	0.231
210	293.5	0.302

(b) The MD and PDI of vesicles incubated in DMEM containing 10% FBS under dark condition were determined by DLS before and after storage for 3 days.

Time (day)	Vesicles	
	MD (nm)	PDI
0	187.8	0.158
3	219.5	0.316

Supplementary Table 2. PQ concentration in zebrafish

PQ concentrations (mean ± SEM, n = 6, determined by LC-MS after exaction of zebrafish tissues) in zebrafish incubated with different formulations for 6 h.

Time (h)	Control	PQ (ng/g body weight)	PQ-loaded vesicles (ng/g body weight)	PQ-loaded vesicles (UV) (ng/g body weight)
1	/	10174.4 ± 198.2	16832.7 ± 242.1	14968.4 ± 212.5
2	/	15386.8 ± 284.6	19040.7 ± 385.3	18301.8 ± 331.8
4	/	19360.1± 394.5	25224.5 ± 5.2.6	25061.2 ± 421.7
6	/	40991.6 ± 946.8	50916.7 ±1026.8	45514.4 ± 984.5
8	/	59525.8 ±1240.5	67919.8 ± 1389.2	65181.6 ± 1364.4

Supplementary Table 3. PQ concentrations in organs

The concentration of PQ in different organs after administration for 30 min and 7 days (Mean ± SEM, n = 6).

Organs	PQ concentration (ng/g tissue)			
	30 min		Day 7	
	PQ	PQ-loaded vesicles	PQ	PQ-loaded vesicles
**Liver**	23042.4±457.2	24182.9±473.4	/	/
**Lung**	9510.5±177.3	9789.8±191.6	/	/
**Kidney**	21228.7±404.6	22529.6±445.3	/	/
**Brain**	/	/	/	/

Supplementary Table 4. Estimation of preparation cost of PQ-loaded vesicles.

(a) The estimated cost for preparing 1 kg CB[8]

Regents	Supplier	Grade	Unit price	Use quantity	Price (CNY)	Price* (USD)
Glycoluril	Alligator Reagent	≥95%	800 CNY/10kg	13.97 kg	1118.53	177.42
Paraformaldehyde	Aladdin	AR	159 CNY/2.5kg	5.89 kg	374.12	57.85
Hydrochloric acid	ACROS ORGANICS	37%, extra pure	639 CNY/5L	19.84 L	2537.91	401.12
Glycerol	Energy Chemical	≥99%	1280 CNY/20L	27.94 L	1789.64	281.56
Methanol	Aladdin	AR, 99.5%	349 CNY/25L	188.99 L	2634.33	416.55
**Total**					**8450.68**	**1334.52**

(b) The estimated cost for preparing 1 kg PQ-loaded vesicles

Regents	Supplier	Grade	Unit price	Use quantity	Price (CNY)	Price* (USD)
CB[8]	/	≥95%	8450 CNY/1kg	672.2 g	5680.09	893.27
PQ	Aladdin	AR	1309 CNY/5g	152 g	39793.6	6258.13
Azobenzene	Aladdin	AR	815 CNY/100g	92.2 g	751.43	118.17
1-bromohexane	Aladdin	AR	167 CNY/500g	83.5 g	27.88	4.38
**Total**					**46253.01**	**7273.95**

Note: The drug loading capacity of PQ-loaded vesicles used in this project was 2.2%.
